# Robotic versus open mesh-enforced retrorectus ventral hernia repair: a multi-center comparative study

**DOI:** 10.1007/s11701-026-03987-1

**Published:** 2026-09-27

**Authors:** Johannes Baur, Marie-Therese Böttcher, Anna Rümmele-Vogt, Michael Meir, Regina Pistorius, Jörg Filser, Beat P. Müller, Daniel C. Steinemann, Fiorenzo V. Angehrn, Julian Süsstrunk, the CROSSFIRE study group

**Affiliations:** 1 Clarunis, Department of Visceral Surgery, University Digestive Health Care Center, St Clara Hospital and University Hospital Basel, Basel, Switzerland; 2 University of Basel, Postfach 4001, Basel, Switzerland; 3 Department of General, Visceral, Transplantation, Vascular and Pediatric Surgery, University Hospital Würzburg, Würzburg, Germany; 4 Department of General, Visceral and Oncologic Surgery, Innklinikum Altötting, Altötting, Germany

**Keywords:** Ventral hernia, Retrorectus hernia repair, Open surgery, Robotic surgery

## Abstract

Mesh-enforced open ventral hernia repair (OVHR) is associated with increased surgical site occurrences and prolonged hospital stay. Robotic-assisted ventral hernia repair (RVHR) may offer benefits compared to OVHR, but comparative literature is scarce. This multicenter prospective cohort study used data from the international CROSSFIRE (MultiCentre InteRnational PrOSpective DatabaSe For Ventral HernIa REpair) register. Patients undergoing OVHR or RVHR for ventral hernias with 3–15 cm transverse diameter between December 2023 and March 2026 were analyzed. The primary outcome was surgical revision after 30 days. Secondary outcomes included postoperative pain after 2 days and 6 weeks, operative time, length of stay (LOS), readmissions and follow-up at 1 year. Two hundred thirty patients were included and analyzed, of which 77 (41% female, BMI 28.4 kg/m2, 90.7% incisional hernias, median hernia diameter 4 cm) underwent OVHR, and 153 (33.7% female, BMI 29.4 kg/m2, 91.7% incisional hernias, median hernia diameter 5 cm) underwent RVHR. Surgical revisions were more frequent after OVHR compared to RVHR (13.7% vs. 4.8%; *p* = 0.027). Median LOS was longer after OVHR (4 days vs. 3days; *p* < 0.001). Median operation time was shorter in OVHR (100 min vs. 210 min; *p* < 0.001). The pain after two days was lower after OVHR (VAS 2 vs. 3; *p* = 0.002); however, it was higher after 6 weeks (2 vs. 1 VAS; *p* = 0.008). After one year, the clinical recurrence rate was higher after OVHR 7.1% vs. 0.0%; *p* = 0.010). RVHR was associated with fewer surgical revisions after 30 days, a shorter LOS, less pain after 6 weeks and improved outcomes after one year. Randomized controlled trials are needed to confirm these findings.

**Supplementary Information:**

The online version contains supplementary material available at https://doi.org/10.1007/s11701-026-03987-1.

## Introduction

Ventral hernia repair is among the most frequently performed surgical procedures in general surgery, with 350.000 to 500,000 procedures performed annually in Europe and the United States [1]. Especially incisional ventral hernias may pose significant clinical challenges, depending on the hernia size and localization, as well as the patient’s individual comorbidities [2].

Mesh-reinforced repair has become the standard of care, as it allows tension-free reconstruction and reduced recurrence compared with primary suture repair [3–5]. However, the optimal mesh position and the best surgical approach for mesh placement remain a matter of ongoing debate.

Open ventral hernia repair has long been considered the gold standard, especially for complex abdominal wall reconstruction, as it allows direct visualization and broad applicability across patient populations, leading to durable outcomes [6]. Despite its proven effectiveness, the open approach is associated with surgical site occurrences, prolonged length of hospital stay, and an increased need for postoperative interventions [7, 8]. Therefore, minimally invasive techniques are increasingly adopted, aiming to reduce those risks.

In recent years, robotic-assisted surgery has emerged as an extended approach of minimally invasive surgery in ventral hernia repair, as the robotic assistance facilitates advanced reconstructive techniques by offering improved visualization and instrument articulation, improved ergonomics, and enhanced dexterity [9]. Conventional laparoscopic techniques have shown significant limitations in achieving retro-rectus mesh reinforcement, as the extensive intracorporal suturing required for posterior sheath and midline fascial closure is technically demanding when performed with non-articulating instruments [10, 11]. The robotic platform overcomes many of these obstacles.

These advantages may translate into improved perioperative outcomes, including reduced complication rates and shorter recovery time.

Despite these potential advantages, high-quality comparative data between robotic and open mesh-enforced ventral hernia repair remains limited and inconclusive. Existing studies are often retrospective, heterogeneous in terms of patient selection and surgical techniques [2, 6, 9, 12].

This study aimed to compare robotic and open mesh-reinforced retrorectus repair with regard to perioperative outcome and follow-up of up to 1 year.

## Methods

### Design and study population

This prospective multicenter cohort study uses data from the international CROSSFIRE (Multi**C**entre Inte**R**national Pr**OS**pective Databa**S**e **F**or Ventral Hern**I**a **RE**pair) database, which incorporates prospective data from the following specialized hernia centers: Altötting Hospital and the University of Würzburg, located in Germany and Visp Hospital, St. Gallen Cantonal Hospital, Limmattal Hospital and the University Digestive Health Care Center- University Hospital Basel and St. Claraspital in Switzerland. This database, along with its collaborating surgeons, is focused on investigating minimally invasive ventral hernia repair techniques and therefore includes all ventral hernias such as primary and incisional hernia. Each surgeon performed at least 30 OVHR and RVHR surgeries. All patients are enrolled consecutively and data collected prospectively at the time of surgery with baseline, perioperative and follow up data entered into the database by the participating centers.(A detailed summary of all collected parameters can be found in the supplementary material). The primary data host is the University Hospital Basel. The study was performed in accordance with the Declaration of Helsinki according to the Strengthening the Reporting of Observational Studies in Epidemiology (STROBE) guidelines [13].

All consecutive adult patients (> 18years) who underwent conventional open or minimally invasive robotic mesh-enforced retrorectus ventral hernia repair between December 2023 and March 2026 at the participating centers, were screened for eligibility. Inclusion criteria entailed a cumulative transverse hernia diameter of 3–15 cm in primary and incisional ventral hernias. Exclusion criteria were the presence of concomitant lateral hernias and severe comorbidities such as liver failure defined as the presence of ascites or renal failure defined as need for renal replacement therapy, as well as emergency surgery and bowel resection.

### Preoperative investigations and surgical procedures

Preoperative investigations comprised a clinical examination of the abdominal wall to identify hernia and rectus diastasis. Ultrasound and computed tomography imaging was obtained at the discretion of the attending surgeon when additional evaluation was considered necessary.

All surgical procedures were performed under general anesthesia, and the patients were placed in a supine position. A single-dose intravenous antibiotic prophylaxis was administered 30–60 min before skin incision, and local anesthetic infiltration was applied at the port sites. In selected patients, epidural anesthesia or transverse abdominal plane block was administered at the discretion of the surgeon/anesthetist.

In the open ventral hernia repair, a midline laparotomy was performed, the hernia sac was resected, and the hernia contents were reduced into the abdominal wall cavity. Adhesiolysis was carried out when necessary. The rectus sheath was opened on both sides under visualization and sparing of the epigastric vessels and neurovascular bundles. Uni- or bilateral transversus abdominis release (TAR) was performed if necessary for closure of the posterior sheath. Mesh augmentation was performed in a retrorectus position using a non-absorbable synthetic mesh, which was fixed using non-absorbable single sutures, glue, or not secured. The anterior fascia was closed using slow-absorbable running sutures.

For the robotic ventral hernia repair, the posterior rectus sheath was accessed with a 12 mm optical port through a subcostal incision. Three 8 mm robotic ports were inserted and the retrorectus space was dissected. Access to the preperitoneal space was gained, and cross-over was performed into the contralateral retrorectus space, as described by Belyanski et al. [14]. The herniated contents were reduced, the hernia sac resected if technically feasible and the posterior plane was closed using running barbed sutures. Uni- or bilateral TAR was performed if necessary to achieve closure of the posterior layer. The hernia defects were closed longitudinally using running absorbable barbed sutures. A non-absorbable synthetic mesh was introduced into the retrorectus space, where it was either fixed with glue or not attached at all.

In both procedures, drain insertion was done at the discretion of the attending surgeon. Patients received standard postoperative analgesia consisting of paracetamol and nonsteroidal anti-inflammatory drugs, as well as prophylaxis for deep vein thrombosis during hospitalization. Upon discharge, the analgesic medication was prescribed on demand. Postoperative follow-up was routinely conducted in the outpatient clinic or, if the patient did not agree to an in-person follow-up visit, with a telephone call 6 weeks after surgery using a personalized link via RedCap. All follow-up visits included questions concerning pain levels after six weeks, the need for pain medication and if sport or work was taken up at precisely what postoperative day. In-person follow-up visits additionally included a thorough clinical examination (inspection and palpation). Imaging was added if either the patient reported a bulge or pain or any symptom considered not normal after one year by the examining clinician. Patients who were followed up through a telephone call but reported symptoms such as abnormal swelling and/or pain were invited for a timely visit at the outpatient clinic. Patients are subsequently followed up yearly until 5 years via letter, email or phone call using a validated hernia follow-up questionnaire including patient-reported measures on perceived hernia recurrence, presence of a visible or palpable bulge, pain and other symptoms at the surgical site [15], .

### Outcome measures

Patient demographics included age, sex, body mass index (BMI) and the presence of relevant comorbidities such as arterial hypertension, type 2 diabetes mellitus, obesity, immunosuppression and the need for anticoagulation. The Comorbidity burden was assessed using the Charlson Comorbidity Index. Hernia-specific variables included hernia size, hernia classification according to the European Hernia Society, Carbonell-Index, and the use of component separation techniques.

The primary outcome was surgical revision within 30 days, which was chosen as a highly objective, clinically relevant endpoint with minimal ascertainment bias that allows direct comparison with existing literature.

Secondary outcomes included pain intensity on postoperative day 2 and at 6 weeks assessed using a visual analog scale (VAS), operative time, and length of stay (LOS). Postoperative morbidity was reported according to the Clavien-Dindo classification and the Comprehensive Complication Index, and the rate of readmission was reported separately [16, 17]. Follow-up was reported using a validated hernia follow-up questionnaire including patient-reported measures on perceived hernia recurrence, presence of a visible or palpable bulge, pain and other symptoms at the surgical site, as well as physician-confirmed hernia recurrence [15].

### Statistical analysis

All data management, statistical analyses, and graphical visualizations were conducted using R (R Foundation for Statistical Computing, Vienna, Austria) within the RStudio integrated development environment (IDE). Continuous variables were assessed for normal distribution using visual inspection (Q-Q plots) and the Shapiro-Wilk test. Variables following a normal distribution were expressed as means with standard deviations (SD), while non-normally distributed continuous variables were presented as medians with interquartile ranges (IQR). Categorical variables were reported as absolute frequencies and percentages.

Baseline patient characteristics, procedural details, and postoperative outcomes were compared between the Open VHR and Robotic VHR groups. For unadjusted analyses, the independent Student’s t-test or the Wilcoxon rank-sum test was utilized for continuous variables as appropriate, while the Chi-squared test or Fisher’s exact test was applied to categorical variables.

To account for baseline confounding and non-randomized treatment assignment, an Inverse Probability Treatment Weighting (IPTW) approach was implemented based on propensity scores. Propensity scores were estimated using a logistic regression model predicting the likelihood of undergoing a robotic approach over an open repair. The model was adjusted for predefined clinical covariates, including hernia transverse diameter, hernia diameter, BMI, age at surgery, hernia type (primary vs. incisional), arterial hypertension, and active smoking status. Weights were stabilized to reduce variance and trimmed at the extreme 5th percentile to prevent undue influence from outlier observations.

The adequacy of the propensity score model and covariate balance achieved by IPTW was rigorously evaluated (**see Figure S1**,** Love Plot**). All statistical tests were two-sided, and a p-value of < 0.05 was considered statistically significant.

## Results

### Patient and hernia characteristics

**Fig. 1:**
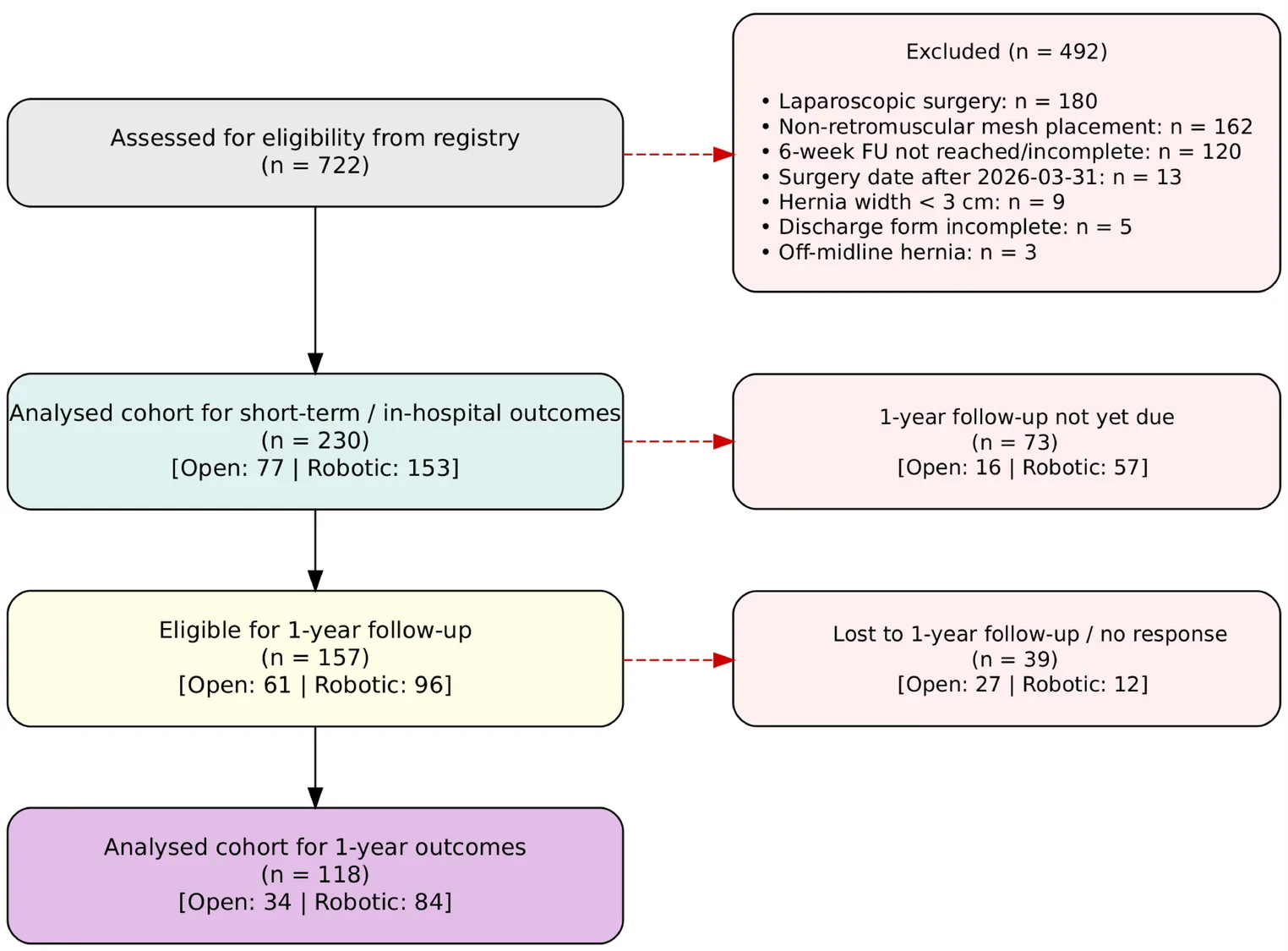
Patient selection flowchart

A total of 230 patients were included in the study consisting of 77 patients in the OVHR group (41% female *p* = 0.310, median age 62 years *p* = 0.554, median BMI 28.4 kg/m2 *p* = 0.659) and 153 patients in the RVHR group (33.7% female, median age 64 years, median BMI 29.4 kg/m2) Further baseline characteristics and the rate of comorbidities were comparable between groups as displayed in Table 1. The selection process is listed in Fig. 1**.**

**Table 1:**
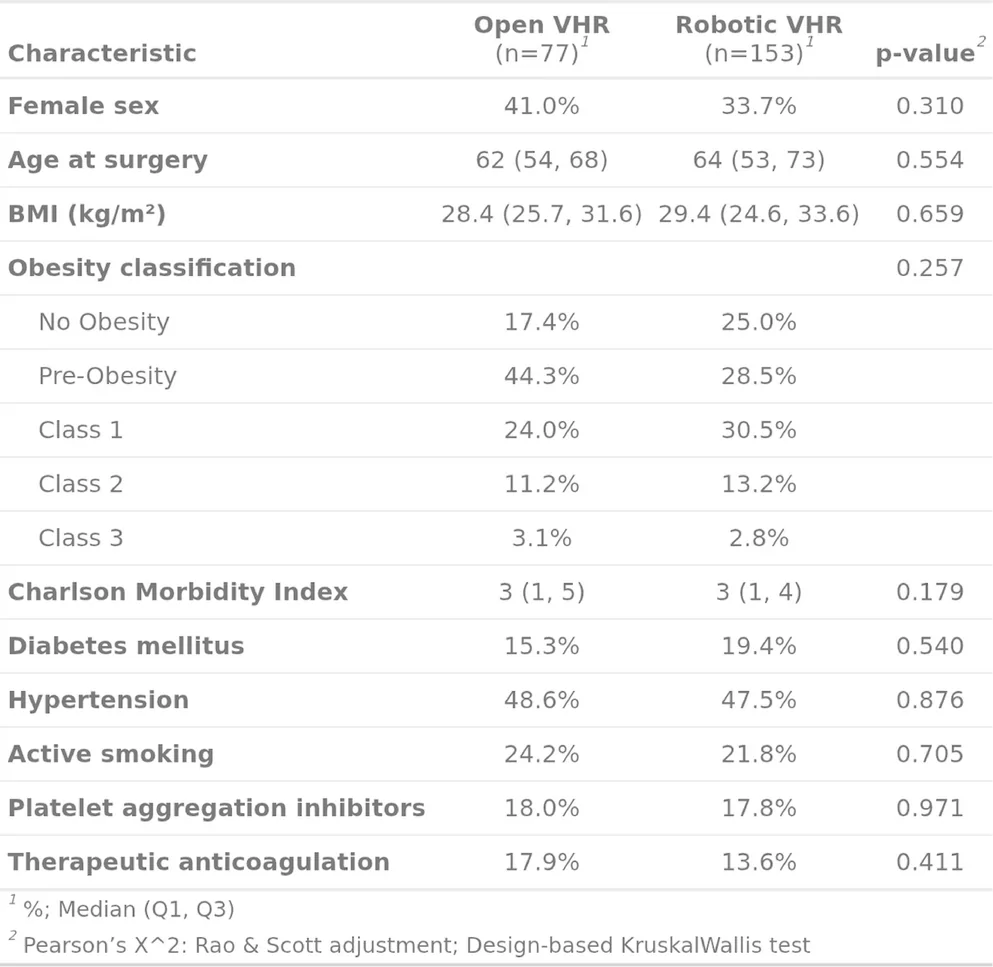
Patient characteristics (adjusted/IPTW weighted)

### Procedural data

More than 90% of hernias were incisional in both groups and hernia characteristics (EHS classification for location/width and median hernia transverse diameter) were comparable among groups (see Table 2). The average OR time was 100 min for OVHR and 210 min for RVHR (*p* < 0.001). The implanted mesh size was larger in the RVHR group (630cm^2^ vs. 370cm^2^; *p* < 0.001), resulting in a larger mesh-defect-ratio (20 vs. 13; *p* = 0.012). Drains were more often placed in the OVHR group compared to the RVHR group (88.1% vs. 5.7%; *p* < 0.001). The form of mesh fixation differed between the two groups (*p* < 0.001); the robotic-assisted repair was more frequently performed without fixation (59.4% vs. 37.1%), but if fixation was used, glue was the main method of fixation in both groups (40.6% vs. 55.4%). Only the open repair group used suture fixation. The rate of TAR was comparable between groups (36.6% in OVHR and 48.7% in RVHR; *p* = 0.096 ).

**Table 2:**
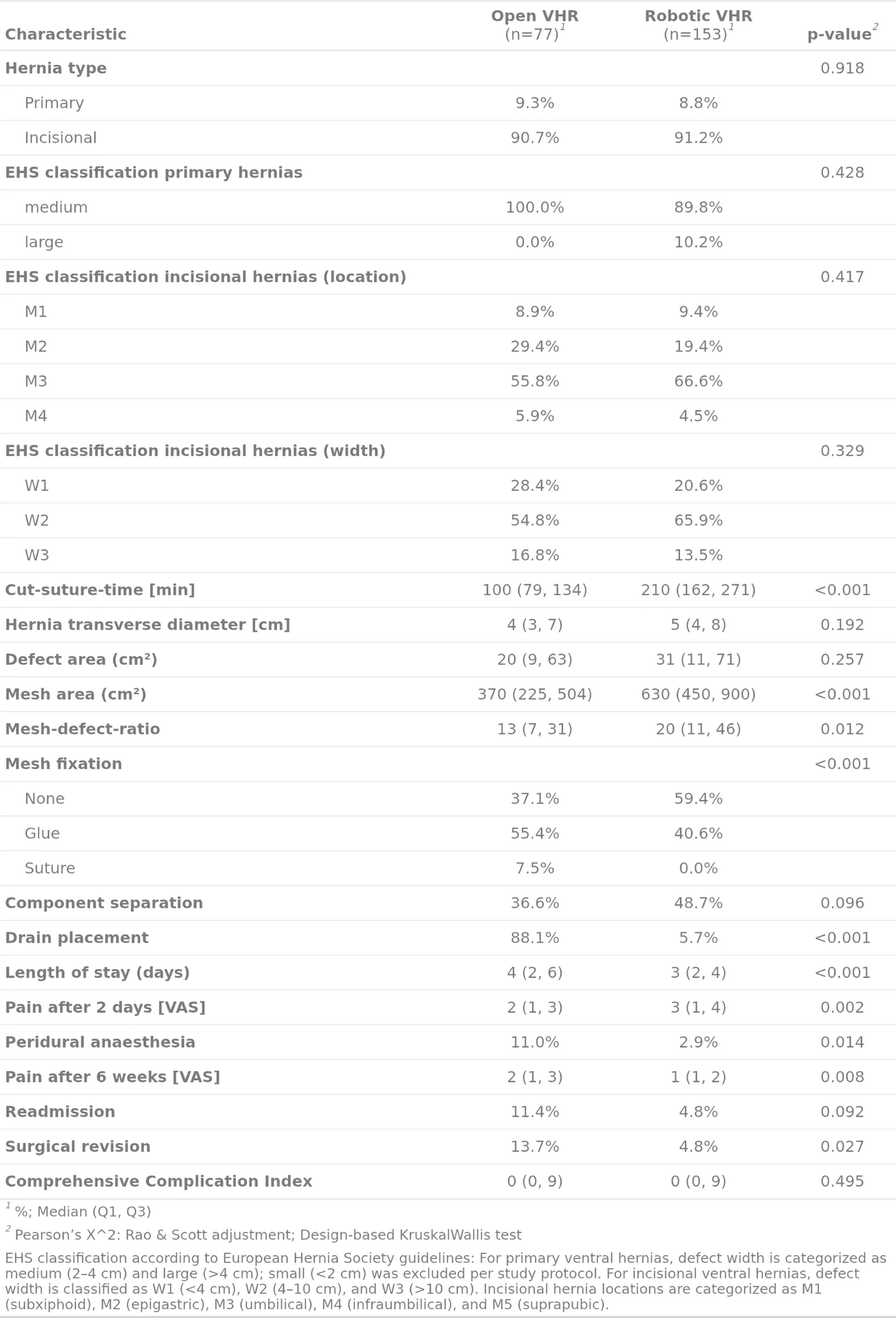
Procedural details and short-term outcomes (adjusted/IPTW weighted)

### Peri- and postoperative outcomes

The rate of surgical revisions within 30 days was significantly higher in the OVHR group (13.7% vs. 4.8%; *p* = 0.027). Five patients required surgical revision within 30 days after RVHR. Indications included open wound revision for surgical site infections (*n* = 2; including one midline infection after prior omphalectomy), minimally invasive revision for bleeding (*n* = 2), and open closure of a trocar hernia (*n* = 1). After OVHR, 11 patients required surgical revision. Reasons for open revision primarily comprised wound complications such as infections and seromas (*n* = 7), as well as bleeding with or without concurrent surgical site infections (*n* = 3). Furthermore, one patient required open revision with small bowel resection due to an intraparietal hernia with rupture of the posterior layer.

**Fig. 2:**
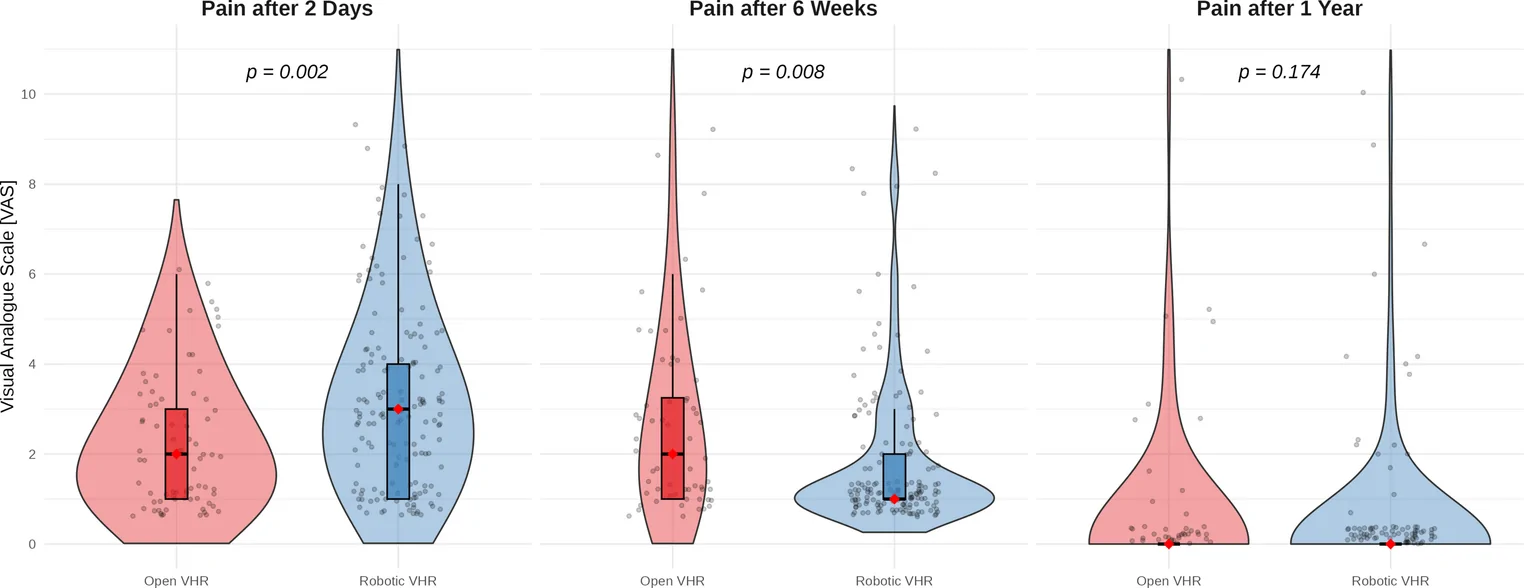
Pain progression (weighted analysis)

**Fig. 3:**
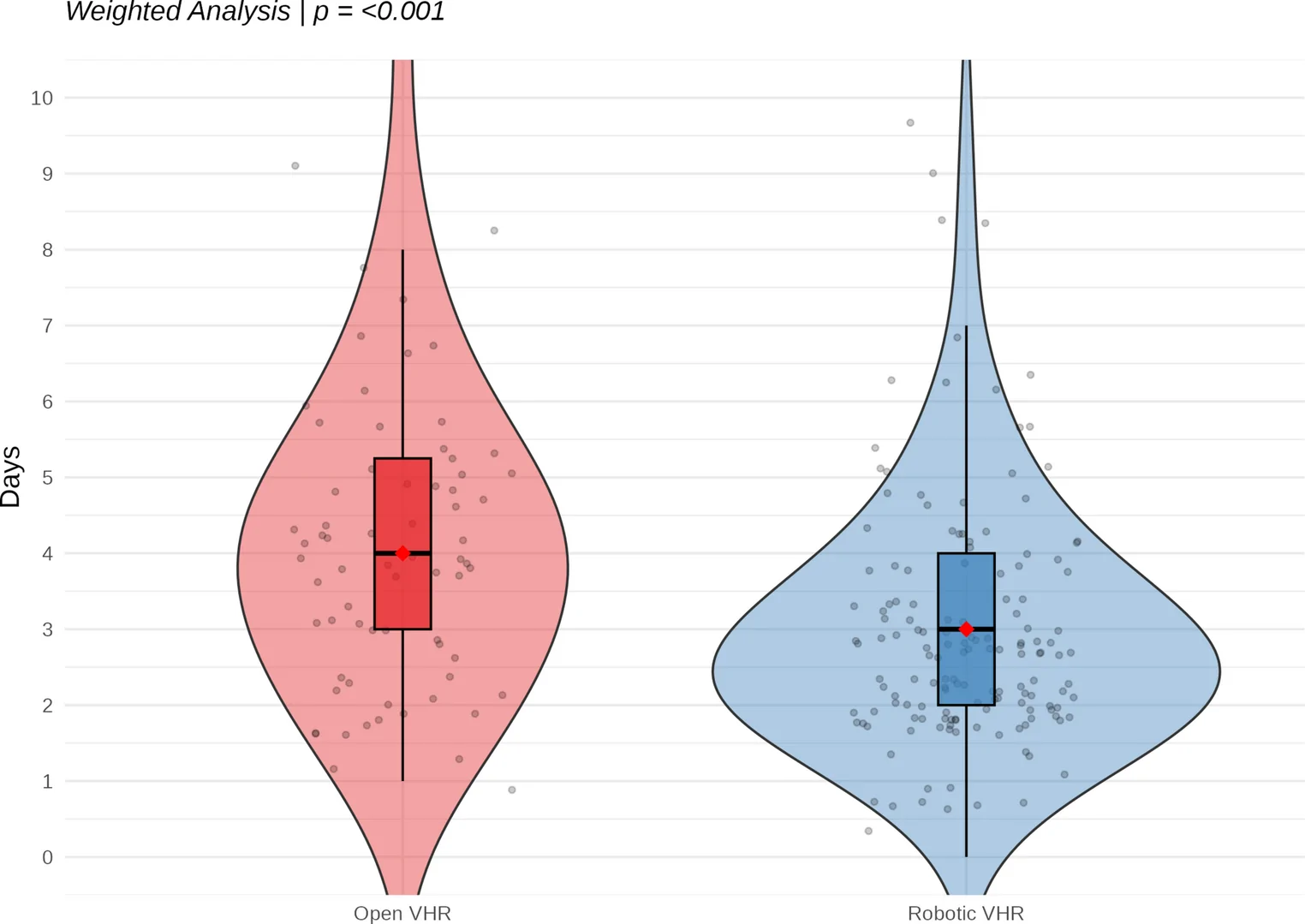
Length of stay (weighted analysis)

The median LOS was longer in OVHR (4 days) compared to RVHR (3 days, *p* < 0.001). The pain score differed significantly between the two groups after 2 days and 6 weeks. After two days, the RVHR group reported higher pain scores compared to the OVHR group (VAS 3 vs. 2; *p* = 0.002); however, after six weeks, the reported pain was lower within the RVHR group (VAS 1 vs. 2; *p* = 0.008). Peridural anesthesia was more often applied in OVHR (11.0% vs. 2.9%; *p* = 0.014). The readmission rate was comparable between groups (11.4% in OVHR vs. 4.8% in RHVR; *p* = 0.092). Table 2; Figs. 2 and 3 provide a detailed overview of procedural details and short-term outcomes.

### One-year follow-up

The overall follow-up rate after one year up to June 2026 was 75.16%. No differences were observed regarding pain outcomes after one year: pain at the surgical site (31.1% OVHR vs. 21.5.% RVHR; *p* = 0.303) and pain intensity after one year (VAS 0 vs. 0; *p* = 0.174) were comparable. More patients reported the presence of a bulge in the OVHR group after one year compared to RVHR (25.2% vs. 9.9%; *p* = 0.037). Patient-reported non-recurrence was lower after OVHR compared to RVHR (72.4% vs. 90.1%; *p* = 0.018). The clinical diagnosis of a recurrence was lower in the RVHR group (0% vs. 7.1%; *p* = 0.010). Table 3 provides detailed information on the one-year outcomes.

**Table 3:**
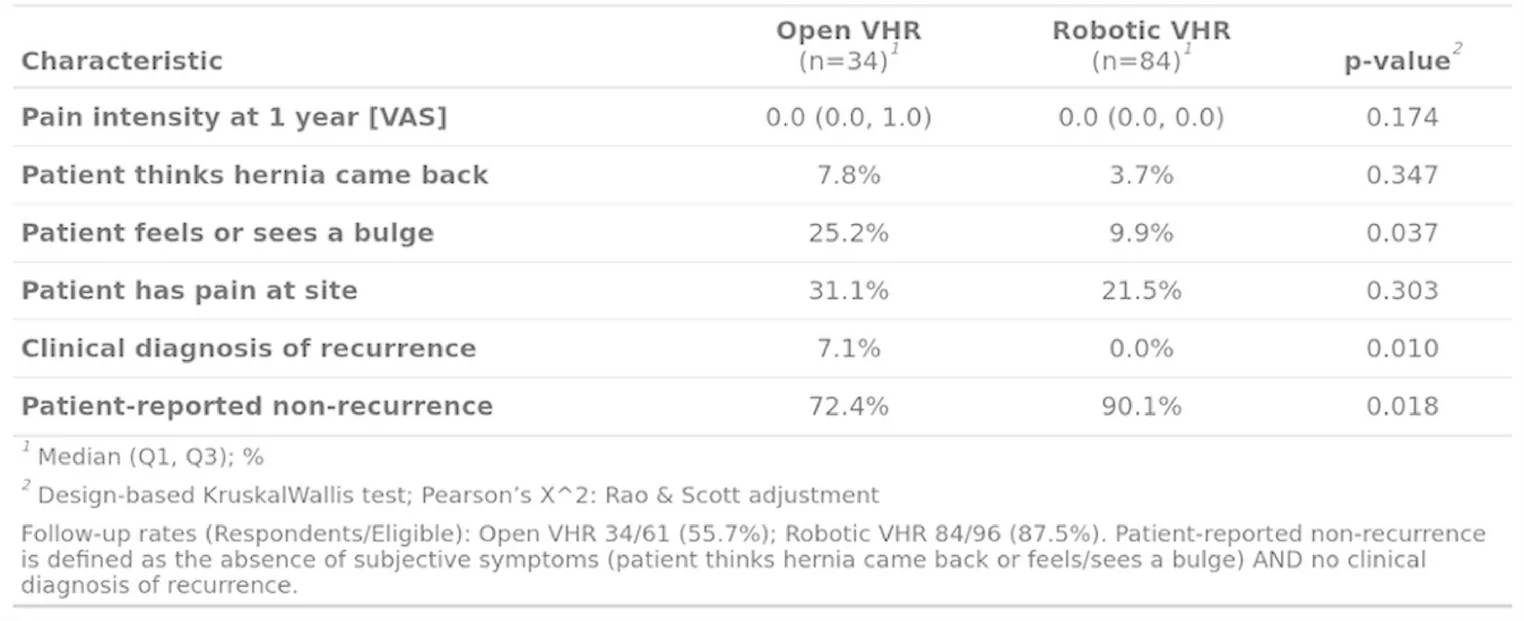
1-Year clinical and patient-reported outcomes (adjusted/IPTW weighted)

## Discussion

The main finding of this multicenter, prospective cohort study is that RVHR leads to fewer surgical revisions within 30 days compared to OVHR. Additionally, RVHR allows insertion of larger meshes even though there are fewer drain placements and a shorter LOS at the cost of a longer procedure time compared to OVHR. After one-year follow-up, the patient reported non-recurrence is higher after RVHR compared to OVHR.

While the baseline transverse hernia diameter and defect area were comparable, the implanted mesh was significantly larger in the RVHR group. This may be explained by the facilitated retro muscular dissection with 3D visualization and favorable ergonomics in RVHR compared to OVHR, where extensive dissection may impose significant physical strain on the surgeon, especially in overweight and obese patients [9]. Although a larger mesh area is dissected in RVHR and fewer drains are used, clinically significant seromas leading to readmission, reinterventions, or reoperations are rare. We assume that every patient undergoing retrorectus repair (open or robotic) develops a seroma as a physiological reaction to this extensive dissection. This seroma, however, is less likely to be clinically relevant in the absence of a midline incision, where infections are more likely to commence and initiate a cascade of infection, fascia rupture and ultimately skin manifestation, thus becoming clinically apparent.

The more extensive dissection in RVHR leads to a larger wound site and may partly explain the higher pain scores 2 days post-surgery (median VAS 3 vs. 2 after RVHR). Furthermore, plication of the entire linea alba, which is mostly performed in RVHR, leads to increased abdominal wall tension and may also have an impact on postoperative pain perception. The ORREO trial by Warren et al. also described higher pain levels after RVHR compared to OVHR (median VAS 3 vs. 2); however, the exact reasons remain unclear [2]. It is important to note that especially the early postoperative pain scores must be interpreted with caution, as more patients in the OVHR received epidural anesthesia or TAP block.

In spite of higher pain scores in RVHR 2 days after surgery, the median LOS was shorter compared to OVHR. This may partly be explained by the less frequent use of epidural anesthesia and drains in RVHR and partly due to a faster general recovery in terms of bowel movements and tolerance of food intake after a minimally invasive procedure. The shorter LOS after RVHR has been observed in several studies comparing open and minimally invasive hernia surgery and showed, similar to our study, that the LOS can be reduced up to 4 days [2, 18–21]. The rate of readmissions was numerically in favor of the RVHR; however, a larger population might be necessary to verify these findings. Drain placement in hernioplasty remains a topic of ongoing debate as the existing literature regarding its benefit, such as less SSI and SSO in general, is controversial [22–24]. In RVHR, we did not see an increased rate of clinically relevant seroma formation despite fewer drain insertions compared to OVHR, and we thus support the omission of drains in RVHR.

Regardless of more pain in the early postoperative days, the patients in the RVHR group showed lower pain levels 6 weeks after surgery, reflecting reduced wound trauma, smaller skin incisions, and reduced soft tissue dissection. Furthermore, the omission of mesh fixation might also contribute to reduced postoperative pain.

The longer operating time in robotic- assisted surgery compared to open surgery has been described in multiple studies up to this date taking up to 49–88 min longer depending on the literature [18–20, 25, 26]. This may be related to the technique itself, as gaining access to the retro muscular space, its dissection and especially the minimally-invasive reconstruction of the linea alba is technically demanding and time-consuming. If bilateral TAR is performed, a double docking is necessary, which also increases operative time, but then allows much further dissection into the preperitoneal lateral space compared to OVHR. We expect the operative time to decrease in the near future, as the technique will be more widely adopted, refined, and mastered within the coming years.

Interestingly, up to this date, the literature on comparing the cost effectiveness of RVHR despite longer operating hours remains conflicting. Recent studies by Sabbatini et al. and Mongelli et al. comparing OVHR vs. RVHR concluded, that in spite of higher costs relating to the surgical procedure, the overall costs were similar after OVHR due to longer hospital-stay costs after OVHR [27, 28]. A study by Kudsi et al. also concluded, that the higher costs after RVHR were balanced by lower post-discharge costs [29]. However, a scoping review by Nielsen et al. comparing 43 studies showed that nonrobotic surgery was 49% less costly and only 22% were in favor of robotic surgery [25]. As mentioned above, there is still a learning curve with robotic surgery and as robotic surgery is used in more complex cases, future studies will show how both factors will influence the total costs.

Our one-year results demonstrate a clear durability benefit for the robotic approach, showing both significantly superior patient-reported outcomes and a complete absence of clinician-diagnosed recurrences in the RVHR group.

Existing literature comparing long-term outcomes after open vs. robotic retorectus hernia repair, however, remains rare and controversial. A recent register study by Remulla et al. comparing OVHR vs. RVHR found higher pragmatic recurrence rates (30.2% vs. 21.3%) three years post-surgery after RVHR despite similar recurrence rates after one and two years. They concluded that this was due to a difference in fascial closure as running fascial closure, which was mainly used in RVHR, was independently associated with high recurrence rates [19].

A significant difference was observed in the subjective perception of a bulge, as patients reported less bulging after RVHR (9.9% vs. 25.2%). This might reflect a more effective abdominal wall reconstruction and reduced scar formation; however, contrary to our study, patients in the ORREO trial reported more bulging after one and two years (16 (41%) vs. 6 (21.2%) and 11 (34.3%) vs. 3 (10%)), though reasons remain unclear in this trial [2]. Similarly, Remulla et al. also reported more bulging after robotic surgery, but concluded that this was due to less skin preclusion after RVHR contributing to the patients’ perceived bulging [19]. However, it is important to note that the sample size differed between the studies as the ORREO trial included 100 patients, our trial included 230 patients and the multicenter matched analysis by Remulla et al. included a total of 2456 patients.

Importantly, the rate of clinically diagnosed recurrence was significantly lower after RVHR, suggesting that the robotic approach may offer greater structural durability of the repair at one year. However, due to the relatively small sample size, particularly in the open repair group, verification with a larger sample size and longer follow-up will be necessary.

To further elucidate the comparative outcomes of OVHR and RVHR, large registry studies, studies with a long follow-up duration, and randomized controlled trials are necessary.

### Limitations

Several limitations need mentioning. First, this study was conducted as a prospective, observational cohort study rather than a randomized controlled trial. This might have led to residual confounding regardless of the prospective nature and applying inverse probability treatment weighting. However, in order to address possible residual confounding and provide a higher level of evidence on this topic, we are planning to conduct a randomized controlled trial investigating OVHR versus RVHR (PRIOR trial: NCT06364306) [30].

Second, the population size is moderate, but comparable to other studies regarding this topic and allowed the detection of clinically relevant differences. Moreover, in this study, follow-up was limited to a one-year follow-up period; however, the patients remain under continued follow-up, which will allow evaluation of long-term clinical outcomes.

Additionally, another relevant limitation was the difference in pain protocol at the participating centers, i.e., use of epidural, TAP block, or opioids. This could lead to a performance bias, thus resulting in higher pain levels in the first postoperative days in the RVHR group.

Moreover, radio-imaging at the one-year follow-up was not performed routinely but rather at the discretion of the clinician, which could have led to under detection and assessment bias in clinical recurrence rates.

The study has several strengths. First, it was designed as a multicenter study, which increases the external validity as the results reflect different surgeons, institutions as well as slightly different patient collectives. Moreover, this design provided a sufficient statistical power to detect less common events, such as surgical revisions. Second, the prospective nature of this study reduces selection bias and allows a standardized and complete data quality, leading to a more precise record of primary and secondary endpoints. Third, the use of IPTW matching reduced confounding bias.

## Conclusion

This prospective comparative study indicates that RVHR offers clinically relevant perioperative benefits compared to an open approach with a lower readmission rate and fewer surgical revisions, as well as a shorter LOS. Furthermore, the pain after 6 weeks was significantly lower in the RVHR in spite of larger mesh implantation. After one year, the clinical recurrence was lower and patient-reported non-recurrence was higher after RVHR. Randomized controlled trials should be initiated to confirm these findings.

## Electronic Supplementary Material

Below is the link to the electronic supplementary material.

Supplementary file

Supplementary material

## Data Availability

All data supporting the findings of this study are available within the paper and its additional material including Tables 1, 2 and 3; Figs. 1, 2 and 3 and S1.
