# Supplementary material for: Robotic versus open mesh-enforced retrorectus ventral hernia repair: a multi-center comparative study
Source: J Robot Surg. 2026 Sep 27;20(1):1029. doi: 10.1007/s11701-026-03987-1 (PMC13616866; doi:10.1007/s11701-026-03987-1)
Supplement: Supplementary file 1 [file 11701_2026_3987_MOESM1_ESM.pdf]

Allgemeine Patientencharakteristika

Bitte füllen Sie folgende Angaben zu Ihrem Patienten aus.

Geschlecht	◯ Männlich<br>◯ Weiblich<br>◯ Divers
Größe in cm	______________________<br>(in cm)
Gewicht in kg	______________________<br>(in kg)
BMI	______________________
Bildgebung vor Diagnose	☐ klinisch<br>☐ Sonographie<br>☐ CT / MRT<br>☐ Zufallsbefund in Bildgebung
Komorbidität (Charlson Comorbidity Index - CCI)	☐ keine<br>☐ Myokardinfarkt (+1)<br>☐ Systolische Herzinsuffizienz (+1)<br>☐ Periphere arterielle Verschlusskrankheit (+1)<br>☐ Zerebrovaskuläre Erkrankung (außer Hemiplegie) (+1)<br>☐ Demenz (+1)<br>☐ Chronische Lungenerkrankung (+1)<br>☐ Kollagenose (+1)<br>☐ Gastroduodenale Ulkuskrankheit (+1)<br>☐ Leichte Lebererkrankung (+1)<br>☐ Diabetes mellitus ohne Endorganschäden (+1)<br>☐ Diabetes mellitus mit Endorganschäden (+2)<br>☐ Hemiplegie (+2)<br>☐ Moderate bis schwere Nierenerkrankung (+2)<br>☐ Tumor (nicht-metastasiert) (+2)<br>☐ Leukämie (+2)<br>☐ Lymphom, Multiples myelom (+2)<br>☐ Moderate bis schwere Lebererkrankung (+3)<br>☐ Metastasierender solider Tumor (+6)<br>☐ AIDS (+6)
Altersgruppe	◯ < 50<br>◯ 50 - 59 (+1)<br>◯ 60 - 69 (+2)<br>◯ 70 - 79 (+3)<br>◯ 80 - 89 (+4)<br>◯ 90 - 99 (+5)<br>(Jahre)
Charlson Comorbidity index	______________________

Performance Status nach WHO-ECOG	◯ 0 - Normale, uneingeschränkte Aktivität, wie vor der Erkrankung<br>◯ 1 - Einschränkung bei körperlicher Anstrengung, gehfähig, leichte körperliche Arbeit möglich<br>◯ 2 - Gehfähig, Selbstversorgung möglich, aber nicht arbeitsfähig, kann mehr als 50% der Wachzeit aufstehen<br>◯ 3 - Nur begrenzte Selbstversorgung möglich; 50% oder mehr der Wachzeit an Bett oder Stuhl gebunden<br>◯ 4 - Völlig pflegebedürftig, keinerlei Selbstversorgung möglich, völlig an Bett oder Stuhl gebunden
Thrombozytenaggregationshemmer (TAH)	◯ Yes<br>◯ No
Therapeutische Antikoagulation (AK)	◯ Yes<br>◯ No
AK Detail	◯ Marcumar<br>◯ Direkte orale Antikoagulanzien (DOAK)<br>◯ andere
AK zeitgerecht pausiert?	◯ Yes<br>◯ No
Marcumar Bridging?	◯ Yes<br>◯ No
Diabetes	◯ kein Diabetes<br>◯ Typ I Diabetes<br>◯ Typ II Diabetes
Hypertension?	◯ keine Hypertonie<br>◯ ein Antihypertonikum<br>◯ zwei antihypertensive Medikamente<br>◯ viele antihypertensive Medikamente
Immunsuppresion	◯ ja<br>◯ nein
Nikotinabusus	◯ ja, aktiv<br>◯ ja, stattgehabt<br>◯ nein

Voroperationen

Speiseröhre ______ Dünndarm ______
Magen ______ Bauchdecke (inkl. Leiste) ______
Leber ______ Herz-/Thoraxchirugie ______
Gallenblase ______ Urologie ______
Pankreas ______ Gynäkologie ______
Kolon/Rektum ______ Gefäßchirurgie ______
Appendix ______ Sonstige ______

Allgemeine eingriffsspezifische Daten

Bitte füllen Sie die eingriffspezifischen Daten zu der erfolgten Operation aus.

Verantwortlicher Chirurg	◯ Baur<br>◯ Angehrn<br>◯ Meir<br>◯ Filser<br>◯ sonstige/sonstiger
Datum der Operation	______________________________
Teaching Eingriff	◯ Yes<br>◯ No
Anteil Teaching	☐ 50% und mehr<br>☐ weniger als 50%
Art der Operation	◯ offen<br>◯ laparo-/endoskopisch<br>◯ robotisch
Netzplatzierung	◯ Sublay (retromuskulär)<br>◯ Präperitoneal<br>◯ Intraperitoneal<br>◯ Onlay
Zugang zur Rektusscheide	◯ transabdominell<br>◯ extraperitoneal
Komponentenseparation erforderlich	◯ Yes<br>◯ No
Art der Komponentenseparation	◯ posterior (TAR)<br>◯ anterior (Ramirez)
Konversion	◯ Yes<br>◯ No
Hybridprozedur	◯ Yes<br>◯ No
primäre vs sekundäre Hernie	◯ primär<br>◯ sekundär
EHS Hernien Klassifikation primäre Hernie	◯ klein (0 -2 cm)<br>◯ medium (2-4 cm)<br>◯ groß (>4cm)
EHS sekundäre Hernie punktum maximum der Hernie	◯ M1<br>◯ M2<br>◯ M3<br>◯ M4<br>◯ M5

Herniengröße	◯ W1<br>◯ W2<br>◯ W3
Rezidiv	◯ Yes<br>◯ No
Nummer Rezidiv	◯ 1.<br>◯ 2.<br>◯ 3.<br>◯ 4.<br>◯ 5.<br>◯ 6.<br>◯ 7,
Anzahl vorhergehende Hernienoperationen	◯ 1<br>◯ 2<br>◯ 3<br>◯ 4<br>◯ 5<br>◯ 6<br>◯ 7
Stattgehabte Hernienoperationen	☐ Direktnaht<br>☐ Sublay<br>☐ IPOM<br>☐ präperitoneales Netz
Begleitende supraumbilikale Rektusdiastase (klinisch oder sonografisch >=3cm)	◯ Yes<br>◯ No
Rektusdiastase therapeutisch adressiert?	◯ nein<br>◯ nur Netzunterfütterung zur Rezidivprophylaxe<br>◯ zusätzliche Raffung
Konkomitante Hernien	☐ nein<br>☐ umbilical<br>☐ epigastrisch
Anzahl konkomitante Hernien	__________________________________
Indikation zur Komponentenseparation	☐ Spannungsfreier Verschluss nicht möglich<br>☐ Unzureichende laterale Überlappung<br>☐ Bruchlücke ausserhalb der Medianen<br>☐ Bruchlücke subxiphoidal<br>☐ Verschluss Präparationsdefekt hintere Schicht
Präoperative Botox-Injektion	◯ Ja<br>◯ Nein
Fasziotens?	◯ Ja<br>◯ Nein
Schnitt-Naht-Zeit (min)	__________________________________

Bruchlückenverschluss - Art	◯ keiner<br>◯ transversal<br>◯ median Bruchpforte<br>◯ median komplett
Bruchlückenverschluss - Fadenmaterial	◯ monophil<br>◯ geflochten<br>◯ Widerhakennaht
Bruchlückenverschluss - Fadenmaterial resorbierbar	◯ Yes<br>◯ No
Bruchlücke - Nahtstärke	◯ 2er<br>◯ 1er<br>◯ 0er<br>◯ 2-0<br>◯ 3-0
Refixierung der Nabelhaut	◯ Yes<br>◯ No
Bruchlücke Querdurchmesser (cm)	__________________________________
Bruchlücke - Längsdurchmesser (cm)	__________________________________
Bruchlückenfläche	__________________________________
Präoperatives CT	◯ Yes<br>◯ No
Breite rechter M. rectus abdominis (cm)	__________________________________
Breite linker M. rectus abdominis (cm)	__________________________________
Carbonell-Index	__________________________________
Netz	◯ PVDF<br>◯ Polypropylene<br>◯ Prolene<br>◯ keines
Netzlänge (cm)	__________________________________
Netzbreite (cm)	__________________________________
Netzfläche	__________________________________

Mesh-Defect-Ratio	__________________
Netzfixierung	☐ keine<br>☐ Kleber<br>☐ Naht resorbierbar<br>☐ Naht nicht resorbierbar<br>☐ transfaszial resorbierbar<br>☐ transfaszial nicht resorbierbar
Drainage Netzlager	◯ ja, aktiv<br>◯ ja, passiv<br>◯ nein
Drainage subkutan	◯ Yes<br>◯ No
Intraoperative Adverse Events	◯ Yes<br>◯ No
Intraoperaitve AE Details	☐ schwer stillbare Blutung<br>☐ Verletzung von Darmstrukturen<br>☐ Konversion<br>☐ Verletzung der Nabelhaut<br>☐ sonstige
Sonstige intraoperative AE	__________________
Nebeneingriffe	◯ Yes<br>◯ No
Nebeneingriff	__________________
Dauer Nebeneingriff (min)	__________________

Outcome bis 6 Wochen postoperativ

Bitte füllen Sie die Nachsorge für den Fragebogen aus.Nachname: [pat_name]
Geburtsdatum: [pat_dob]
Patienten:ID [pat_id]

Datum der Entlassung

____

Aufenthaltsdauer

____

Schmerzen 2. postoperativer Tag oder zum Zeitpunkt der Entlassung

◯ 0
◯ 1
◯ 2
◯ 3
◯ 4
◯ 5
◯ 6
◯ 7
◯ 8
◯ 9
◯ 10

Zug letzte Drainage (POT)

____

Datum 1. Nachsorge

____

Zeitpunkt Nachsorge (d nach OP)

____

Schmerzscore 1. Nachsorge

◯ 0
◯ 1
◯ 2
◯ 3
◯ 4
◯ 5
◯ 6
◯ 7
◯ 8
◯ 9
◯ 10

Werden noch Schmerzmittel eingenommen?

◯ Yes
◯ No

Patient treibt regelmässig Sport (bereits präoperativ)

◯ Yes
◯ No

Patient hat mit sportlicher Tätigkeit postoperativ wieder begonnen?

◯ Yes
◯ No

Wiederbeginn Sport (Tage nach OP)

____

Patient berufstätig?

◯ Yes
◯ No

Hat der Patient die berufliche Tätigkeit postoperativ wieder aufgenommen?	◯ Yes<br>◯ No
Wiederaufnahme Arbeit (d nach OP)	__________________________________
Postoperative Komplikation	◯ Yes<br>◯ No
Ausserplanmässige ambulante Vorstellung?	◯ Yes<br>◯ No
Grund ambulante Vorstellung	☐ Schmerzen<br>☐ Serom<br>☐ Hämatom<br>☐ Wundsekretion<br>☐ Wundinfektion<br>☐ sonstiges
Wiederaufnahme nach Entlassung	◯ Yes<br>◯ No
Postoperative Intervention	◯ Yes<br>◯ No
Dauer des stationären Aufenthaltes nach Wiederaufnahme (d)	__________________________________
Operative Revision	◯ Yes<br>◯ No
Postoperative Kompliaktion Detail	☐ Nachblutung<br>☐ Dehiszenz Inzision<br>☐ Infekt Inzision<br>☐ Schaden Nabelhaut<br>☐ Infektion Nabelhaut<br>☐ Schaden sonstige Haut<br>☐ Infektion sonstige Haut<br>☐ Netzinfekt<br>☐ Netzausbau<br>☐ Verletzung intraabdomineller Organe<br>☐ Nahtriss Bruchlückenverschluss<br>☐ intraparietale Hernie<br>☐ Dysästhesie<br>☐ persistierende Schmerzen<br>☐ Serom<br>☐ Ileus<br>☐ Pneumonie<br>☐ Tiefe Venenthrombose<br>☐ Lungenembolie<br>☐ sonstige
Sonstige Komplikation	__________________________________

Seromklassifikation

- ◯ Typ I: Klinisches Serom weniger als 1 Monat nachweisbar
- ◯ Typ II: Klinisches Serom mehr als 1 Monat nachweisbar
- ◯ Typ III: Minor-Komplikation des Serom (mehr als 6 Monate, Schmerz...)
- ◯ Typ IV: Major-Komplikationen des Seroms die ein Behandlung erfordern

---

Klassifikation nach Clavien-Dindo

Grad I ______
Grad II ______
Grad IIIa ______
Grad IIIb ______
Grad IVa ______
Grad IVb ______
Grad V ______

Comprehensive Complication Index (CCI) ______________________________

Comprehensive Complication Index (CCI) ______________________________

CCI Calculator

NASA Task Load

Bitte füllen Sie den NASA-Task-Load für den Patienten aus.

1) Tag des Ausfüllens

____

2) Mentale Anforderung

Wie mental anspruchsvoll war die Aufgabe?

sehr niedrig — sehr hoch

(Place a mark on the scale above)

3) Physische Anforderung

Wie physisch anspruchsvoll war die Aufgabe?

sehr niedirg — sehr hoch

(Place a mark on the scale above)

4) Zeitliche Anforderung

Wie eilig oder gehetzt war das Gangart der Aufgabe?

sehr niedrig — sehr hoch

(Place a mark on the scale above)

5) Performance

Wie erfolgreich waren Sie bei der Erfüllung dessen, was Sie zu tun hatten?

Perfekt — Versagen

(Place a mark on the scale above)

6) Aufwand

Wie hart mussten Sie arbeiten, um das Leistungsniveau zu erreichen?

sehr wenig — sehr viel

(Place a mark on the scale above)

7) Frustration

Wie verunsichert, entmutigt, irritiert, gestresst, und genervt waren Sie?

sehr wenig — sehr stark

(Place a mark on the scale above)

EuraHS Qu Post OP

Vielen Dank, dass Sie sich bereit erklärt haben an unserer Patientenbefragung teilzunehmen.

Schmerzen im Bereich des OP Gebietes

1) Durchschnittlicher Schmerz in Ruhe

Auf einer Skala von 0 - 10 wobei
0 = keine Schmerzen
10 = maximale Schmerzen

- ◯ 0
- ◯ 1
- ◯ 2
- ◯ 3
- ◯ 4
- ◯ 5
- ◯ 6
- ◯ 7
- ◯ 8
- ◯ 9
- ◯ 10

2) Durchschnittlicher Schmerz bei Belastung / Bewegung

Auf einer Skala von 0 - 10 wobei
0 = keine Schmerzen
10 = maximale Schmerzen

- ◯ 0
- ◯ 1
- ◯ 2
- ◯ 3
- ◯ 4
- ◯ 5
- ◯ 6
- ◯ 7
- ◯ 8
- ◯ 9
- ◯ 10

3) Stärkster Schmerz innerhalb der letzten Woche

Auf einer Skala von 0 - 10 wobei
0 = keine Schmerzen
10 = maximale Schmerzen

- ◯ 0
- ◯ 1
- ◯ 2
- ◯ 3
- ◯ 4
- ◯ 5
- ◯ 6
- ◯ 7
- ◯ 8
- ◯ 9
- ◯ 10

Restriktion von Alltagsakivitäten durch die Operation

4) Wie stark belastet sie die Operation der Hernie bei täglichen Aktivitäten im Haushalt

Auf einer Skala von 0 - 10 wobei
0 = keine Behinderung
10 = maximale Behinderung

- ◯ 0
- ◯ 1
- ◯ 2
- ◯ 3
- ◯ 4
- ◯ 5
- ◯ 6
- ◯ 7
- ◯ 8
- ◯ 9
- ◯ 10
- ◯ nicht ausgeführt

5) Wie stark belastet sie die Operation der Hernie bei täglichen Aktivitäten (Spazierengehen, Autofahren, gemütliches Fahrradfahren)

Auf einer Skala von 0 - 10 wobei
0 = keine Behinderung
10 = maximale Behinderung

- ◯ 0
- ◯ 1
- ◯ 2
- ◯ 3
- ◯ 4
- ◯ 5
- ◯ 6
- ◯ 7
- ◯ 8
- ◯ 9
- ◯ 10
- ◯ nicht ausgeführt

6) Wie stark belastet sie die Operation der Hernie beim Sport?

Auf einer Skala von 0 - 10 wobei
0 = keine Behinderung
10 = maximale Behinderung

- ◯ 0
- ◯ 1
- ◯ 2
- ◯ 3
- ◯ 4
- ◯ 5
- ◯ 6
- ◯ 7
- ◯ 8
- ◯ 9
- ◯ 10
- ◯ nicht ausgeführt

7) Wie stark belastet sie die Operation der Hernie bei schwerer körperlicher Tätigkeit

Auf einer Skala von 0 - 10 wobei
0 = keine Behinderung
10 = maximale Behinderung

- ◯ 0
- ◯ 1
- ◯ 2
- ◯ 3
- ◯ 4
- ◯ 5
- ◯ 6
- ◯ 7
- ◯ 8
- ◯ 9
- ◯ 10
- ◯ nicht ausgeführt

Kosmetische Beeinträchtigung

8) Wie empfinden Sie die Form Ihres Bauches insgesamt nach der Operation?

Auf einer Skala von 0 - 10 wobei
0 = Sehr schön
10 = sehr hässlich

- ◯ 0
- ◯ 1
- ◯ 2
- ◯ 3
- ◯ 4
- ◯ 5
- ◯ 6
- ◯ 7
- ◯ 8
- ◯ 9
- ◯ 10

9) Wie empfinden Sie die Kosmetik im Bereich der ehemaligen Hernie

Auf einer Skala von 0 - 10 wobei
0 = Sehr schön
10 = sehr hässlich

- ◯ 0
- ◯ 1
- ◯ 2
- ◯ 3
- ◯ 4
- ◯ 5
- ◯ 6
- ◯ 7
- ◯ 8
- ◯ 9
- ◯ 10

EuraHS Qu Prä OP

Vielen Dank, dass Sie sich bereit erklärt haben an unserer Patientenbefragung teilzunehmen.Bitte füllen Sie nun die Fragen zu Ihrer Lebensqualität vor dem geplanten Eingriff aus.

Schmerzen im Bereich der Hernie

1) Durchschnittlicher Schmerz in Ruhe

Auf einer Skala von 0 - 10 wobei
0 = keine Schmerzen
10 = maximale Schmerzen

- ◯ 0
- ◯ 1
- ◯ 2
- ◯ 3
- ◯ 4
- ◯ 5
- ◯ 6
- ◯ 7
- ◯ 8
- ◯ 9
- ◯ 10

2) Durchschnittlicher Schmerz bei Belastung / Bewegung

Auf einer Skala von 0 - 10 wobei
0 = keine Schmerzen
10 = maximale Schmerzen

- ◯ 0
- ◯ 1
- ◯ 2
- ◯ 3
- ◯ 4
- ◯ 5
- ◯ 6
- ◯ 7
- ◯ 8
- ◯ 9
- ◯ 10

3) Stärkster Schmerz innerhalb der letzten Woche

Auf einer Skala von 0 - 10 wobei
0 = keine Schmerzen
10 = maximale Schmerzen

- ◯ 0
- ◯ 1
- ◯ 2
- ◯ 3
- ◯ 4
- ◯ 5
- ◯ 6
- ◯ 7
- ◯ 8
- ◯ 9
- ◯ 10

Restriktion von Alltagsakivitäten

4) Wie stark belastet sie die Hernie bei täglichen Aktivitäten im Haushalt

Auf einer Skala von 0 - 10 wobei
0 = keine Behinderung
10 = maximale Behinderung

- ◯ 0
- ◯ 1
- ◯ 2
- ◯ 3
- ◯ 4
- ◯ 5
- ◯ 6
- ◯ 7
- ◯ 8
- ◯ 9
- ◯ 10
- ◯ nicht ausgeführt

5)	Wie stark belastet sie die Hernie bei täglichen Aktivitäten (Spazierengehen, Autofahren, gemütliches Fahrradfahren)<br><br>Auf einer Skala von 0 - 10 wobei<br>0 = keine Behinderung<br>10 = maximale Behinderung	◯ 0<br>◯ 1<br>◯ 2<br>◯ 3<br>◯ 4<br>◯ 5<br>◯ 6<br>◯ 7<br>◯ 8<br>◯ 9<br>◯ 10<br>◯ nicht ausgeführt
6)	Wie stark belastet sie die Hernie beim Sport?<br><br>Auf einer Skala von 0 - 10 wobei<br>0 = keine Behinderung<br>10 = maximale Behinderung	◯ 0<br>◯ 1<br>◯ 2<br>◯ 3<br>◯ 4<br>◯ 5<br>◯ 6<br>◯ 7<br>◯ 8<br>◯ 9<br>◯ 10<br>◯ nicht ausgeführt
7)	Wie stark belastet sie die Hernie bei schwerer körperlicher Tätigkeit<br><br>Auf einer Skala von 0 - 10 wobei<br>0 = keine Behinderung<br>10 = maximale Behinderung	◯ 0<br>◯ 1<br>◯ 2<br>◯ 3<br>◯ 4<br>◯ 5<br>◯ 6<br>◯ 7<br>◯ 8<br>◯ 9<br>◯ 10<br>◯ nicht ausgeführt

Kosmetische Beeinträchtigung

8)	Wie empfinden Sie die Form Ihres Bauches insgesamt?<br><br>Auf einer Skala von 0 - 10 wobei<br>0 = Sehr schön<br>10 = sehr hässlich	◯ 0<br>◯ 1<br>◯ 2<br>◯ 3<br>◯ 4<br>◯ 5<br>◯ 6<br>◯ 7<br>◯ 8<br>◯ 9<br>◯ 10

9) Wie empfinden Sie die Vorwölbung der Hernie

Auf einer Skala von 0 - 10 wobei
0 = Sehr schön
10 = sehr hässlich

◯ 0
◯ 1
◯ 2
◯ 3
◯ 4
◯ 5
◯ 6
◯ 7
◯ 8
◯ 9
◯ 10
